# Radiological progression of COVID‐19 organizing pneumonia

**DOI:** 10.1002/rcr2.764

**Published:** 2021-05-04

**Authors:** Bushra Johari, Roqiah Fatmawati Abdul Kadir, Farihah Abd Ghani, Alan Basil Peter

**Affiliations:** ^1^ Faculty of Medicine Universiti Teknologi MARA Sungai Buloh Malaysia; ^2^ Department of Radiology Hospital Sungai Buloh Sungai Buloh Malaysia

**Keywords:** Computed tomography, COVID‐19, organizing pneumonia, X‐ray

## Abstract

It has recently been recognized that the clinical course of coronavirus disease 2019 (COVID‐19) and secondary organizing pneumonia (OP) tend to follow a subacute progression of respiratory illness. We present images of radiological progression of COVID‐19 pneumonia and secondary OP.

## Graphical abstract

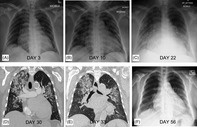

## Clinical Image

**Figure 1:**
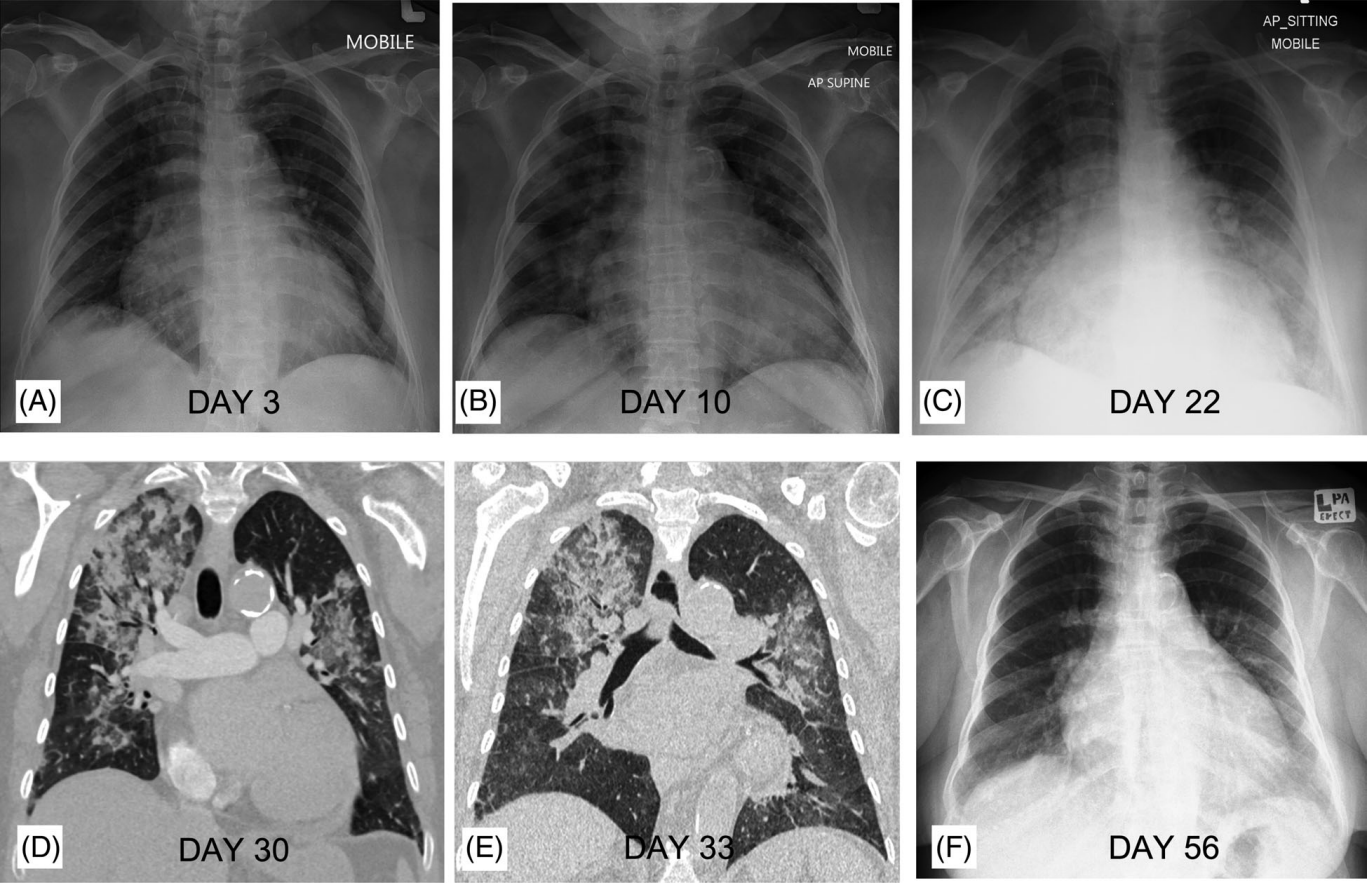
Chest X‐ray (CXR; A–C and F) and coronal reconstruction of computed tomography (CT) in lung window (D, E). (A) Day 3 of coronavirus disease 2019 (COVID‐19) diagnosis: no evidence of lung consolidation. (B) Day 10: bilateral peripheral consolidations. (C) Day 22: bilateral mid to lower zone consolidations which appeared more confluent. (D) Day 30: perilobular consolidations surrounding ground‐glass opacities in keeping with organizing pneumonia (OP). (E) Day 33: OP started to improve following recommencement of steroid therapy. (F) Day 56: resolved lung changes.

A 73‐year‐old lady with multiple medical comorbidities presented with cough and tested positive for coronavirus disease 2019 (COVID‐19). Her symptoms worsened at day 10 where she desaturated to 80% under room air requiring high‐flow mask oxygenation. Chest X‐ray (CXR) showed bilateral peripheral consolidation. She was given intravenous methylprednisolone (200 mg for four days followed by 150 mg for two days) and subsequently 8 mg dexamethasone. Her supplemental oxygen requirement ceased and she showed symptomatic improvement following treatment; thus she was discharged at day 17 with tapering dose of oral prednisolone. She re‐presented again to the hospital at day 22 with dyspnoea. CXR revealed bilateral confluent consolidation and computed tomography (CT) at day 30 revealed changes typical of organizing pneumonia (OP) (Fig. 1). Cultures for other infections were negative. She was again treated with steroids (oral prednisolone 30 mg once daily for two weeks) and was discharged well from the ward 39 days after her initial diagnosis. The steroid dose was progressively tapered down over three months' time (Fig. 1). It has recently been recognized that the clinical course of COVID‐19 and secondary OP tend to follow a subacute progression of respiratory compromise. These patients have been called “happy hypoxics,” with low oxygen levels in the absence of respiratory distress, thus secondary OP might be missed by those unfamiliar with this entity [1]. In some cases, antiviral therapy was instituted [2]. Re‐administration of steroid improved our patient's clinical condition and radiological findings, similar to other recently reported cases [3, 4]. Pattern recognition of OP on CT is vital as it can help expedite proper management, in the absence of histopathological tissue diagnosis. The dose and duration of steroid treatment for secondary OP need to be tailored carefully, as these patients often need higher doses and longer treatment duration [1].

### Disclosure Statement

Appropriate written informed consent was obtained for publication of this case report and accompanying images.

### Author Contribution Statement

Bushra Johari carried out the literature review and wrote the manuscript draft. Roqiah Fatmawati Abdul Kadir contributed to the manuscript revision and figure preparation. Farihah Abd Ghani and Alan Basil Peter collected the case information and contributed to manuscript revision. All authors read and approved the final manuscript.
